# The natural course of low back pain from childhood to young adulthood – a systematic review

**DOI:** 10.1186/s12998-018-0231-x

**Published:** 2019-03-20

**Authors:** Tina Junge, Niels Wedderkopp, Eleanor Boyle, Per Kjaer

**Affiliations:** 1 Department of Sports Science and Clinical Biomechanics, University of Southern Denmark, Campusvej 55, 5230 Odense, Denmark; 2 Health Sciences Research Centre, University College Lillebaelt, Odense, Denmark; 3 Department of Orthopaedics, Sydvestjysk Sygehus Esbjerg, Esbjerg, Denmark

**Keywords:** Natural course, Low back pain, Children and adolescence

## Abstract

**Background:**

Taking the natural course of recurrent and fluctuating low back pain (LBP) seen in longitudinal studies of adults into consideration, the aetiology and development of LBP in children and adolescents also needs to be reflected in a long-term course. Therefore, a systematic critical literature review was undertaken to assess the natural course of LBP in the general population from childhood through adolescence to young adulthood.

**Methods:**

A systematic literature search was conducted in MEDLINE, EMBASE, CINAHL and PsycINFO with synonyms of search terms for 1) low back pain; 2) natural course; 3) cohort study and 4) children. Records in English, German, French, Danish, Swedish, and Norwegian were included. To assess the methodological quality of the studies, the NIH quality assessment checklist for cohort studies was adapted and risk of bias was assessed on a study level. Two authors independently reviewed selected studies, assessed quality, and extracted data. A synthesis of results in relation to the natural course of LBP was created.

**Results:**

Totally, 3373 records were identified, eight articles were included for quality assessment, and finally, four studies of good to fair quality were included for synthesis of results. Indication of three common patterns of LBP were identified across studies and labelled as 1) ´children and adolescents with no LBP or low probability of LBP´ (49 to 53%), 2) ´children and adolescents with fluctuation of LBP´ (16 to 37%) and 3) ´children and adolescents with repeated reporting of LBP´ (< 1 to 10%).

**Conclusion:**

Although methodological heterogeneity, mainly due to different age ranges, an indication of a natural course of LBP was seen across studies. The majority of children and adolescents repeatedly reporting no or low probability of LBP. With recall periods between one week to three months and sampling rates ranging from one to four years, a very low rate repeatedly reported LBP, and approximately one-fifth to one-third of children and adolescents had fluctuating reports of LBP. A need of future research of LBP trajectories with short reporting period lengths and narrower sampling windows in a long-term perspective is emphasized in order to study childhood influences on the development of LBP throughout life.

**Electronic supplementary material:**

The online version of this article (10.1186/s12998-018-0231-x) contains supplementary material, which is available to authorized users.

## Background

Although the majority of children and adolescents report absence of pain, the prevalence of self-reported low back pain (LBP) increases throughout adolescence and reaches adulthood levels at the age of 18 years [1, 2]. The reported prevalence rates vary in the literature from 0.8 to 84% [1, 2], which is dependent upon the definition of LBP, the age group assessed, the method for collecting information, and the type of prevalence reported. An increase of LBP prevalence with age is seen in children and adolescents, indicating that these are the decades in life, in which any vulnerability develops or becomes apparent [3].

Nevertheless, while prevalence studies in cross-sectional studies present the proportion of the population reporting LBP at a certain time point, within a certain period or ever, it is not possible to determine from these studies whether it is the same or different children or adolescents, who report LBP at different ages and time points, seen in a long-term perspective [3]. As prevalence studies of LBP only describes the population-averaged status of LBP, and hence does not reflect the development or course of LBP, they provide limited information about the condition with respect to portrayal of health care consequences and prevention strategies.

In adults, longitudinal cohort studies have provided an understanding of the development or course of individual pain conditions over time with respect to later health or disease risk, indicating that some people experience pain throughout their lives. In adults, there is consensus of the natural course of LBP, demonstrated as either being a persistent or episodic condition with recurrent events rather than one well-defined episode or episodes of unrelated occurrence [3–5]. This fluctuating and recurrent nature might also be evident in children and youth and track into adulthood; therefore, the aetiology and development of LBP in children and adolescents should be considered in a long-term course with frequent data collection points [6]. To our knowledge, no systematic summary of the natural course of LBP exists in children and adolescents.

The ability to identify and describe the natural course or time-based progressions of distinctive clusters of variation in LBP, called developmental trajectories, could be an important aid to improve the understanding of development and changes of LBP status over time. The identified trajectories might be able to provide unique information on LBP and its impact during the transition from childhood to adolescence to young adulthood, and to provide a means of exploring certain risk factors and detecting groups of frail children who are particularly susceptible for developing LBP.

Therefore, taking the fluctuating and recurrent natural course of LBP status in adults into consideration [3, 5], the natural course of LBP in children and adolescents should also be considered and described in a long-term course with frequent measurements of LBP [6].

The aim was to study the natural course of LBP in the general population from childhood through adolescence to young adulthood using a systematic critical literature review.

## Methods

### Search strategy

The systematic literature search was conducted in MEDLINE via PubMed, EMBASE, CINAHL complete and PsycINFO in the period of September 2018 to November 2018, with the following search terms: ´back pain´, ´spinal pain´ or ´neck pain´; ´natural course´, ´natural history´, ´trajectories´, ´transitional´, ´tracking´, ´prognostic´, ´prediction´, ´patterns´ or ´follow-up´; cohort study´, ´epidemiological study´ or ´longitudinal study´; ´children´, ´infants´, ´youth´, ´teenagers´ or ´adolescence´, ´adolescents´, ´young adulthood´ or ´young adults´. References in the following languages; English, German, French, Danish, Swedish and Norwegian were included. The search protocol was refined in collaboration with a university librarian. Search terms, in- and exclusion criteria were adapted to the databases, and the references from the search result were exported to Covidence; an online software product for managing systematic reviews [7]. Duplets were reviewed, reported and excluded in this program and validated by reviewing the reference list in Endnote as well. A full electronic search strategy for the search in PubMed is applied in Additional file 1. The searches were re-run just before the final analyses and further studies retrieved for inclusion. The protocol of the current systematic review was registered in PROSPERO, registration number CRD42018111000.

### Inclusion and exclusion criteria

Inclusion criteria for the systematic review were studies concerning: 1) low back pain 2) description or analysis of the natural course of low back pain, with a period of follow-up with at least two data collection time points, taking into account the individual status of LBP between time points, and 3) a general population of children and adolescents with a cohort including either a period from childhood to adolescence or adolescence to adulthood (maximum 22 years). The main outcome was low back pain in children and adolescents, measured over time. Exclusion criteria were studies only reporting prevalence or incidence of LBP; intervention studies; clinical or working populations; specific sub groups of back disorders such as scoliosis; and adolescents above the age of 18 at baseline.

### Review process

**Fig. 1:**
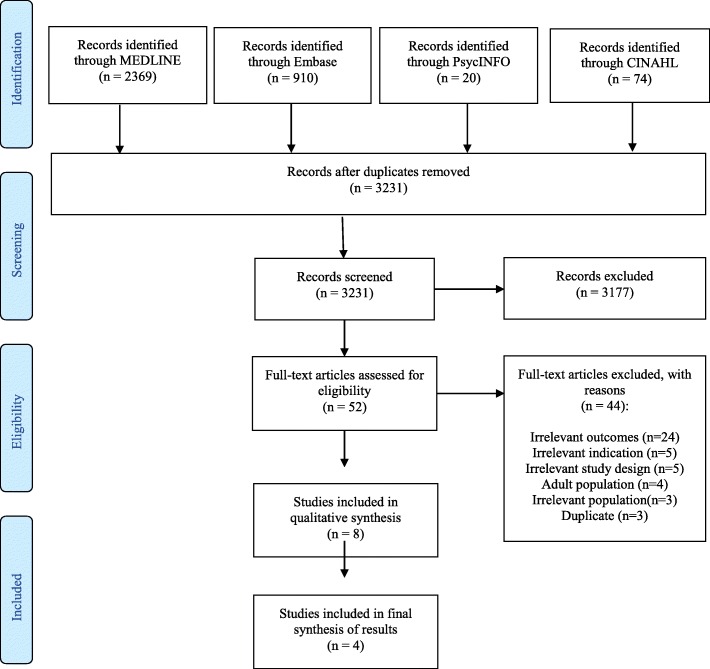
PRISMA flow diagram The flow diagram maps out the number of records identified from four databases, the included and excluded studies, and the reasons for study exclusions

Based on the predefined inclusion and exclusion criteria, two of the authors independently assessed the eligibility of potential studies. Disagreements were resolved either through discussion of the studies or by involvement of a third author. For the articles that PK and NW were authors on, TJ and EB were the team that independently assessed the eligibility, performed quality assessment and data extraction. The numbers of studies searched and included, and the reasons for study exclusion are presented as a flow diagram (Fig. 1). The excluded studies are listed in Additional file 2.

### Quality assessment

Two of the authors independently reviewed the included studies. To assess the methodological quality of the studies, an adapted version of the National Institute of Health (NIH) quality assessment tool for observational cohort and cross-sectional studies was applied and customized to the review question of interest [8]. Questions related to exposure were only judged in relation to how the reporting of previous LBP was used as a determinant for reporting of LBP during the current time-frame. The final determination of the quality of the selected studies was based on whether the included studies had minimized potential bias in their study design, and was rated as good, fair or poor, based on the NIH quality assessment and potential risk of bias due to selection and misclassification bias, outcome data bias, selective outcome reporting and other potential sources of bias.

Good and fair studies were included in the synthesis of results in relation to describing the natural course of LBP, which is presented in narrative text, tables, and figures. Due to heterogeneity of outcome measurements, it was not possible to perform meta-analysis.

## Results

In total, 3373 records were located. Following removal of duplicates, 3231 abstracts were screened, and 52 full-text studies were assessed for eligibility. For various reasons, listed in the PRISMA flow diagram in Fig. 1, 44 studies were excluded.

### Quality assessment

A total of eight articles were included in the current review for quality assessment (Aartun et al., Burton et al., Coenen et al., Grimmer et al., Kjaer et al., Mikkelsson et al., Sjolie et al., Szpalski et al.) [9–16]. Characteristics of the studies are presented in Table 1. An overview of assessment of quality is presented in Table 2.

**Table 1:** Overview of the included studies for quality assessment

Author and year	Study design and setting	Age of cohort at baseline	Purpose of study	Number of follow-ups	Time between follow-ups	Sample size	Methods for data collection	Outcome measures of LBP	Identified terminology for patterns of LBP
Aartun, 2014	School-based prospective cohort study	11–13 years	Course of LBP	1	2 years	Baseline: 1291 Follow-up: 1064 (82%)	Electronic questionnaire	1) Point prevalence 2) 1-week prevalence 3) Lifetime prevalence	1) Never LBP 2) Development of LBP 3) Changes in frequency of LBP
Burton, 1996	School-based prospective cohort study	11 years	Natural history of LBP	4	1 year	Baseline: 216 Follow-up (T5): 147 (68%)	Questionnaire; interviews first 2 years, then self-administered	1) Lifetime prevalence	1) Single, discrete spell of LBP 2) Recurrent LBP
Coenen, 2017	Follow-up in a birth cohort	17 years	Trajectories of LBP	2	3 and 2 years	Baseline: 1050 Follow-up (T2): 1033 (98%) (not same responders T1–3)	Self-administered questionnaire	1) 1 month prevalence	1) Consistenly low probability of LBP 2) Increase in prevalence of LBP 3) Decrease in the prevalence of LBP 4) Consistently high prevalence of LBP
Grimmer, 2006	School-based prospective cohort study	13 years	Tracking of LBP	4	Yearly samples	Baseline: 434 Follow-up (T5): 174 (40%)	Self-administered questionnaire	1) 1-week prevalence	1) Recent LBP 2) Variability of LBP 1) Repeated reporting 2) Regular LBP 3) Consistency of occurrence of LBP
Kjaer, 2011	School-based prospective cohort study	9 years	Tracking of LBP	3	4 years	Baseline: 479 Follow-up: 443 (92%) (not same responders T1–4)	Questionnaire by interviews	1) Point prevalence 2) 1-week prevalence 3) 1 month prevalence	1) No LBP 2) Still LBP 3) Changes in reports of LBP
Mikkelsson, 1997	School-based prospective cohort study	9 years	Persistence of LBP	1	1 year	Baseline: 1756 Follow-up:1628 (92%)	Self-administered questionnaire	1) 3 months prevalence	1) Seldom or never LBP 2) Persistence of LBP
Sjolie, 2004	School-based prospective cohort study	14 years	Persistence and change in LBP	1	3 years	Baseline: 88Follow-up: 85 (97%)	Self-administered questionnaire	1) 1-year prevalence	1) No LBP 2) Still LBP 3) Persistent, but changeable LBP
Szpalski, 2002	School-based prospective cohort study	9 years	Prediction of LBP	1	2 years	Baseline: 287 Follow-up: 287 (100%)	Self-administered questionnaire	1) Lifetime prevalence	1) No LBP 2) Identical LBP 3) Ongoing LBP

**Table 2:** NIH quality assessment for cohort studies

Author	Aartun	Burton	Coenen	Grimmer	Kjaer	Mikkelsson	Sjolie	Szpalski
1. Was the research question or objective in this paper clearly stated?	Y	Y	Y	Y	Y	Y	Y	Y
2. Was the study population clearly specified and defined?	Y	Y	CD	Y	Y	Y	Y	Y
3. Was the participation rate of eligible persons at least 50%?	Y	Y	Y	Y	Y	Y	Y	Y
4. Were all the subjects selected or recruited from the same or similar populations (including the same time period)? Were inclusion and exclusion criteria for being in the study prespecified and applied uniformly to all participants?	Y	N	Y	Y	N	Y	Y	N
5. Was a sample size justification, power description, or variance and effect estimates provided?	N	N	N	Y	N	N	N	N
6. For the analyses in this paper, were the exposure(s) of interest measured prior to the outcome(s) being measured?	Y	Y	Y	Y	Y	Y	Y	Y
7. Was the timeframe sufficient so that one could reasonably expect to see an association between exposure and outcome if it existed?	N	N	Y	Y	Y	N	Y	N
8. For exposures that can vary in amount or level, did the study examine different levels of the exposure as related to the outcome (e.g., categories of exposure, or exposure measured as continuous variable)?	NA	NA	NA	NA	NA	NA	NA	NA
9. Were the exposure measures (independent variables) clearly defined, valid, reliable, and implemented consistently across all study participants?	Y	N	Y	Y	Y	Y	Y	N
10. Was the exposure(s) assessed more than once over time?	N	N	Y	Y	Y	N	N	N
11. Were the outcome measures (dependent variables) clearly defined, valid, reliable, and implemented consistently across all study participants?	Y	Y	Y	N	N	Y	Y	N
12. Were the outcome assessors blinded to the exposure status of participants?	NA	NA	NA	NA	NA	NA	NA	NA
13. Was loss to follow-up after baseline 20% or less?	Y	N	Y	N	N	Y	Y	Y
14. Were key potential confounding variables measured and adjusted statistically for their impact on the relationship between exposure(s) and outcome(s)?	N	N	N	N	N	N	N	N

*CD*, cannot determine; *NA*, not applicable

### Risk of bias

In the study by Aartun et al., several risks of bias were suspected due to differential drop-outs, missing sensitivity analyses and problems with baseline data collection. High reporting of life time prevalence due to questionnaire technique affected the results of prevalence, change, and course of LBP. No statistics were applied for analysis of course or changes of LBP; these could be the same children or new cases. The analyses performed did not support the conclusion. Overall, the quality of the study was rated as poor (Table

3).

Several risks of bias were considered in the study by Burton et al. due to no inclusion or exclusion criteria, applying a not validated questionnaire with the risk of reporting bias for lifetime prevalence of LBP. Different types of data collection methods were seen over time, with interviews at the first two time points followed by self-reported questionnaires. Also, a large dropout rate was reported. Overall, the quality of the study was rated as poor (Table 3).

**Table 3:** Overview of risk of bias

	AARTUN	Burton	Coenen	Grimmer	Kjaer	Mikkelsson	Sjolie	Szpalski
Overall rating	Poor	Poor	Good	Fair	Fair	Fair	Poor	Poor
Risk of bias	Reporting bias Misclassification bias Differential drop outs Analytical bias Selective outcome reporting	Selection bias Reporting bias Misclassification bias Loss to follow-up	Selection bias	Misclassification bias Loss to follow-up Analytical bias	Selection bias Reporting bias Misclassification bias	Reporting bias Misclassification bias	Reporting bias Misclassification bias Analytical bias	Selection bias Reporting bias Misclassification bias Analytical bias Selective outcome reporting

In the study by Coenen et al., few risks of bias were detected, mainly due to no inclusion or exclusion criteria and dropouts in the profiling analyses. Overall, the quality of the study was rated as good (Table 3).

Some risk of bias was seen in the study by Grimmer et al. with a large dropout rate, particularly in the end of the study, and also, a small study sample, which did not qualify for the many statistical analyses. Overall, the quality of the study was rated as fair (Table 3).

In the study by Kjaer et al., some risk of bias was found since no validated questionnaire was applied. Outcome measure was collapsed to one dichotomous answer of the presence of back pain from reports of point, one-week and one-month questions of back pain. Also, no inclusion or exclusion criteria was listed, and a large dropout was seen. Overall, the quality of the study was rated as fair (Table 3).

Some risk of bias was also considered in the study by Mikkelsson et al. since the study sample was small for children with persistent LBP (*n* = 64 first time point, *n* = 22 s time point). The cohort was assessed twice with selected reporting of results of solely one-week prevalence of LBP. No statistical analysis was applied to answer the research question of persistence of LBP. Overall, the quality of the study was rated as fair (Table 3).

In the study by Sjolie et al., several risks of bias were considered due to a small study sample, with a risk of type 2 error for prediction models. Also, reporting bias was suspected due to questions of one-year prevalence of LBP. Overall, the quality of the study was rated as poor (Table 3).

Several risks were considered in the study by Szpalski et al. as no inclusion or exclusion criteria was listed, no validated questionnaire was applied, and also, reporting bias was suspected due to lifetime prevalence of LBP. Reporting of results was only from children responding at both time points. The non-significant results could indicate a type 2 error of results due to a small sample size. Overall, the quality of the study was rated as poor (Table 3).

In summary, one study was rated as good (Coenen et al.) and three studies as fair (Grimmer et al., Kjaer et al., Mikkelsson et al.). All four studies were included for synthesis of results, as summarized in Table 3.

### Synthesis of results

Diverse terms for describing the natural course of LBP was seen across studies although no definitions of the various terms were provided. For synthesis of results, the associated terms of 1) ´no LBP´ or ´low probability of LBP´ were collapsed in one column, 2)´variability of LBP´, ´ increase of LBP´, ´decrease of LBP´, and ´changing tracking pattern´ were labelled as ´fluctuation of LBP´, and 3) high prevalence of LBP´, ´consistently reporting LBP´, and ´persistence of LBP´ were labelled as ´repeated reporting of LBP´ (Table 4).

**Table 4:** Overview of results from the included studies

	Age range of participants	No or low probability of LBP	Repeated reporting of LBP	Fluctuation of LBP
Coenen	17–22	53%	10%	37%
Grimmer	13–17	–	< 1%	16%
Kjaer	9–15	49%	< 1%	32%
Mikkelsson	9–11	–	1,3%	–

In the study by Coenen et al., focusing on the 17 to 22-year olds, four clusters were identified for describing trajectories of LBP and its impact from adolescence to young adulthood: 1) consistently low probability of LBP (53%), 2) increase in LBP (22%), 3) decreasing LBP (15%), and 4) consistently high prevalence of LBP (10%) with indicator variables including six variables (one for LBP and five additional impact items) at each of the three time points (Table 4).

Assessing the 13 to 17-year olds, Grimmer et al. found variability in LBP reporting between study years for 16% of the children. Also, consistently reporting of LBP was noticed, although the numbers are small; two children reported recent LBP every study year, and three children in the last four study years, accounting for less than 1% of the study population (Table 4).

In the study by Kjaer et al., including the 9 to 15-year olds, < 1% repeatedly reported LBP at all time points, 49% reported no LBP at all time points (*n* = 261, participating at all time points) (Table 3). For those not participating in all three surveys, 32% of children with LBP (first time point) and 38% of the children with LBP (second time point) reported LBP at the next time point. This was only 1.4 and 8% of all participants at the second and third time point, respectively. Extracting the drop outs (*n* = 118), the percentages were equal to 2 and 11%. Having reported LBP at one time point compared to not having reported LBP, increased the probability of reporting pain again in the next time point; thereby, increasing with age. Amongst responders, 19% reported LBP in a changing tracking pattern between first and second time points, while it was 28% from second to third time points. Subtracting the drop outs from first time point to second time point (n = 118), the total amount of children reporting LBP in a changing tracking pattern were 26 and 38%, respectively (Table 4).

In the 9 to 11-year olds, who were studied by Mikkelsson et al., one-year persistence of pain at least once a week was seen in 34% of the children; at baseline, 64 children (4%) reported LBP as opposed to follow-up, with 22 children (1.3%) reporting LBP at least once a week again. The results were not related to school grade. One-year changes in musculoskeletal pain symptoms were not stratified in pain location areas such as LBP (Table 4).

## Discussion

In this systematic review, a total of four studies of good to fair quality were included for studying the natural course of LBP from childhood to young adulthood. An indication of a common pattern of LBP was seen across the studies, although methodological heterogeneity, mainly due to different age ranges. The majority of the children and adolescents repeatedly reported no or low probability of experiencing LBP. With recall periods between one week to three months and sampling rates ranging from one to four years, a small proportion of the children and adolescents repeatedly reported having LBP during the study period, whereas one-fifth to one-third of the children and adolescents had fluctuating reports of LBP.

To our knowledge, this is the first review to assess evidence of the natural course of LBP in the life course stage of childhood to young adulthood. A few studies of good to fair quality were included in the current review, and despite heterogeneity, mainly due to different age spans and prevalence measures, some indication of a similar course was seen across studies. The most common reporting in children and adolescents is no LBP [4], and as seen in the current review, it is also the most common reporting over time. Also, very low rates of repeated reporting of LBP was seen across studies, increasing with age. Interestingly, fluctuating reporting of LBP was also commonly seen, with periods of pain and periods with absence of pain, although dissimilar terms such as ´variability´ or ´changes´ were used across studies. Standardisation of a terminology for labelling courses or principal trajectory patterns might be useful in future research to describe findings in standardised ways [5].

The findings of the current study are comparable to analyses of trajectories of musculoskeletal pain including back pain in 11 to 14-year olds, with the majority (78%) of the study sample having a cluster of ´no pain problem´ throughout follow-up, other clusters were fluctuating in reports (total of 23%), and finally, a very small cluster (1.3%) had very high probability of pain throughout follow-up [17]. The indications of a natural course of LBP in children and adolescents seen in the current review have some similarities to the course of LBP described in adults, where most people recover quickly from new episodes of low back pain and recurrence or fluctuation is common, and in only a small proportion, LBP becomes persistent and disabling [3–5].

Consensus of a natural course of LBP in children and adolescents is inconclusive from this review, and indeed, more life-course epidemiological studies are needed to explore the natural course or trajectories of LBP from childhood to young adulthood, as well as to assess predictors of the course by elucidating the specific influence of the timing, nature and duration of LBP episodes on future health [3, 5]. Specifically, identifying and describing trajectories among children and adolescents could improve understanding of how pain conditions like LBP can develop and fluctuate over time [17]. For a reliable estimation of inter-individual variability in intra-individual patterns of change over time, such as in trajectory models, an adequate sample size of at least 100 individuals is needed, typically requiring at least three repeated measures per individual, although the number of data points needed for trajectory analyses depends on the intended level of detail [5, 18]. Using multiple measurement points in longitudinal studies has advantages over simpler approaches of defining outcome at a single time point, as seen in earlier studies of courses and development of LBP from childhood to adulthood [19–21].

When assessing self-reported musculoskeletal health in children over time, there are also several other concerns to be made. Except for one study [13], where health professionals performed interviews with the children, all studies provided self-reported questionnaires of LBP. Only few studies applied validated questionnaires, although these were not tested for feasibility in children or tested for reliability [9, 11, 15]. The stability of measurement properties over time are crucial with respect to assessment of the natural course of musculoskeletal health, and it is for this reason, self-reported questionnaires must be designed for and tested on the specific age group, with special emphasis on the reporting period. In more studies of the current review, it was found that up to 60% of the children who reported one-year or a lifetime history of LBP at one timepoint did not report the same at the next timepoint [9, 10, 15, 16]. In line with others, these findings indicate that experiences of LBP often are common, short-lasting and benign of nature [4], hence not creating a memorable impact for the single child or adolescent; therefore, one-year and lifetime prevalence numbers of LBP for this population can have limited value. This is in accordance with others, recommending the avoidance of long recall periods as this may cause reporting bias [22]. Further, to assess the ´true prevalence´ of LBP future studies need to use multiple repeated measurements over time, at least every month, or even every week, to minimize reporting bias.

The strengths of the current systematic review are the specific search terms related to the research question, the independent assessment of eligibility, quality assessment, data extraction, and the synthesis of results of only good to fair quality studies. The limitation of the current systematic review is, on the other hand, the narrow search terms used, which may exclude some relevant studies. It can be questioned, if a search of the broader term ´musculoskeletal pain´ in children and adolescents could have been more informative and valuable, as it has been argued that trajectories are similar across musculoskeletal pain conditions, such as back pain, headache, and facial pain in adolescents, and in LBP and knee pain in adults [5, 17]. However, summarised findings from a large, prospective cohort study of school children with weekly assessments of musculoskeletal pain in children and youth indicates this is not the case [23–26].

## Conclusion

Four studies of good to fair quality were included for studying the natural course of LBP from childhood to young adulthood. Although methodological heterogeneity, mainly due to different age ranges, an indication of a natural course of LBP was seen across the studies. The majority of the children and adolescents repeatedly reported no or low probability of experiencing LBP. A small proportion of the children and adolescents repeatedly reported having LBP within recall periods of one week to three months and sampling rates ranging from one to four years, whereas one-fifth to one-third of the children and adolescents had fluctuating reports of LBP. A need of future research of LBP trajectories with short reporting period lengths and narrower sampling windows in a long-term perspective is emphasized in order to study childhood influences on the development of LBP through life.

## Additional files

Additional file 1: in PDF format: Search strategy for MEDLINE via PubMed. The detailed search strategy for MEDLINE via PubMed is an example of the search strategy for this database. (PDF 33 kb)

Additional file 2: in PDF format: List of excluded studies. The studies excluded during the eligibility phase are listed in Additional file 2. (PDF 76 kb)

## Abbreviations

LBP: Low back pain
NIH: National Institute of Health
