# Supplementary material for: The natural course of low back pain from childhood to young adulthood – a systematic review
Source: Chiropr Man Therap. 2019 Mar 20;27:10. doi: 10.1186/s12998-018-0231-x (PMC6425623; doi:10.1186/s12998-018-0231-x)
Supplement: Supplementary file 1 — in PDF format: Search strategy for MEDLINE via PubMed. The detailed search strategy for MEDLINE via PubMed is an example of the search strategy for this database. (PDF 33 kb) [file 12998_2018_231_MOESM1_ESM.pdf]

Additional file 1. Search strategy for MEDLINE via PubMed

(back pain OR neck pain OR spinal pain) AND (natural course OR natural history OR trajectories OR Trajectory OR Tracking OR Transitional OR Prognostic OR Prediction OR Predictive OR Patterns OR Follow-up) AND (children OR infants OR teenagers OR adolescence OR adolescents OR youth OR young adulthood OR young adults) AND (cohort study OR epidemiological study OR longitudinal study)
