# Supplementary material for: The natural course of low back pain from childhood to young adulthood – a systematic review
Source: Chiropr Man Therap. 2019 Mar 20;27:10. doi: 10.1186/s12998-018-0231-x (PMC6425623; doi:10.1186/s12998-018-0231-x)
Supplement: Supplementary file 2 — in PDF format: List of excluded studies. The studies excluded during the eligibility phase are listed in Additional file 2. (PDF 76 kb) [file 12998_2018_231_MOESM2_ESM.pdf]

Additional file 2. List of excluded studies

1. Auvinen, J. P., Paananen, M. V., Tammelin, T. H., Taimela, S. P., Mutanen, P. O., Zitting, P. J., & Karppinen, J. I. (2009). Musculoskeletal pain combinations in adolescents. Spine (Phila Pa 1976), 34(11), 1192-1197.
2. Ahacic, K., & Kreholt, I. Prevalence of musculoskeletal pain in the general Swedish population from 1968 to 2002: Age, period, and cohort patterns. Pain. 2010 Oct;151(1):206-14.
3. Barnekow-Bergkvist, M., Hedberg, G. E., Janlert, U., & Jansson, E. (1998). Determinants of self-reported neck-shoulder and low back symptoms in a general population. Spine (Phila Pa 1976), 23(2), 235-243.
4. Borchgrevink, P. C., & Rustoen, T. The prevalence of recurrent pain in childhood is high and increases with age. Scand J Pain. 2018 Jul 26;18(3):341-342.
5. Brattberg, G. (2004). Do pain problems in young school children persist into early adulthood? A 13-year follow-up. Eur J Pain, 8(3), 187-199.
6. Croft, P. R., Lewis, M., Papageorgiou, A. C., Thomas, E., Jayson, M. I., Macfarlane, G. J., & Silman, A. J. (2001). Risk factors for neck pain: a longitudinal study in the general population. Pain, 93(3), 317-325.
7. Dissing, K. B., Hestbaek, L., Hartvigsen, J., Williams, C., Kamper, S., Boyle, E., & Wedderkopp, N. Spinal pain in Danish school children - How often and how long? the CHAMPS Study-DK. BMC Musculoskelet Disord. 2017 Mar 27;18(1):67.
8. Dunn, K. M., Jordan, K. P., Mancl, L., Drangsholt, M. T., & Le Resche, L. Trajectories of pain in adolescents: A prospective cohort study. Pain. 2011 Jan; 152(1): 66–73.
9. El-Metwally, A., Salminen, J. J., Auvinen, A., Macfarlane, G., & Mikkelsson, M. (2007). Risk factors for development of non-specific musculoskeletal pain in preteens and early adolescents: a prospective 1-year follow-up study. BMC Musculoskelet Disord, 8, 46.
10. Feldman, D. E., Rossignol, M., Shrier, I., & Abenhaim, L. Smoking: a risk factor for development of low back pain in adolescents. American Journal of Epidemiology, Volume 154, Issue 1, 1 July 2001, Pages 30–36.
11. Gustafsson, M. L., Laaksonen, C., Aromaa, M., Loyttyniemi, E., & Salantera, S. (2018). The prevalence of neck-shoulder pain, back pain and psychological symptoms in association with daytime sleepiness - a prospective follow-up study of school children aged 10 to 15. Scand J Pain, 18(3), 389-397.
12. Hertzberg, A. (1985). Prediction of cervical and low-back pain based on routine school health examinations. A nine- to twelve-year follow-up study. Scand J Prim Health Care, 3(4), 247-253.

13. Hestbaek, L., Korsholm, L., Leboeuf-Yde, C., & Kyvik, K. O. (2008). Does socioeconomic status in adolescence predict low back pain in adulthood? A repeated cross-sectional study of 4,771 Danish adolescents. Eur Spine J, 17(12), 1727-1734.
14. Hestbaek, L., Leboeuf-Yde, C., Kyvik, K. O., & Manniche, C. (2006). The course of low back pain from adolescence to adulthood: eight-year follow-up of 9600 twins. Spine (Phila Pa 1976), 31(4), 468-472.
15. Hestbaek, L., Leboeuf-Yde, C., & Kyvik, K. O. (2006). Is comorbidity in adolescence a predictor for adult low back pain? A prospective study of a young population. BMC Musculoskelet Disord, 7, 29.
16. Hestbaek, L., Leboeuf-Yde, C., & Kyvik, K. O. (2006). Are lifestyle-factors in adolescence predictors for adult low back pain? A cross-sectional and prospective study of young twins. BMC Musculoskelet Disord, 7, 27.
17. Incledon, E., O'Connor, M., Giallo, R., Chalkiadis, G. A., & Palermo, T. M. Child and Family Antecedents of Pain During the Transition to Adolescence: A Longitudinal Population-Based Study. J Pain. 2016 Nov;17(11):1174-1182.
18. Janssens, K. A. M., Rosmalen, J. G. M., Ormel, J., Verhulst, F. C., Hunfeld, J. A. M., Mancl, L. A., . . . LeResche, L. Pubertal Status Predicts Back Pain, Overtiredness, and Dizziness in American and Dutch Adolescents. Pediatrics. 2011 Sep; 128(3): 553–559.
19. Johansson, M. S., Jensen Stochkendahl, M., Hartvigsen, J., Boyle, E., & Cassidy, J. D. (2017). Incidence and prognosis of mid-back pain in the general population: A systematic review. Eur J Pain, 21(1), 20-28.
20. Jones, G. T., Johnson, R. E., Wiles, N. J., Chaddock, C., Potter, R. G., Roberts, C., . . . Macfarlane, G. J. (2006). Predicting persistent disabling low back pain in general practice: a prospective cohort study. Br J Gen Pract, 56(526), 334-341.
21. Jones, G. T., Watson, K. D., Silman, A. J., Symmons, D. P. M., & Macfarlane, G. J. Predictors of low back pain in British schoolchildren: a population-based prospective cohort study. Pediatrics. 2003 Apr;111(4 Pt 1):822-8.
22. Kroner-Herwig, B., Gorbunova, A., & Maas, J. (2017). Predicting the occurrence of headache and back pain in young adults by biopsychological characteristics assessed at childhood or adolescence. Adolesc Health Med Ther, 8, 31-39.
23. Larsson, B., & Sund, A. M. Emotional/behavioural, social correlates and one-year predictors of frequent pains among early adolescents: Influences of pain characteristics. Eur J Pain. 2007 Jan;11(1):57-65.

24. Leino-Arjas, P., Rajaleid, K., Mekuria, G., Nummi, T., Virtanen, P., & Hammarstrom, A. Trajectories of musculoskeletal pain from adolescence to middle age: The role of early depressive symptoms, a 27-year follow-up of the Northern Swedish Cohort. Pain. 2018 Jan;159(1):67-74.
25. Lourenco, S., Correia, S., Alves, L., Carnide, F., Silva, S., & Lucas, R. (2017). Intergenerational educational trajectories and lower back pain in young women and men. Acta Reumatol Port, 42(1), 73-81.
26. Mikkelsson, M., El-Metwally, A., Kautiainen, H., Auvinen, A., Macfarlane, G. J., & Salminen, J. J. (2008). Onset, prognosis and risk factors for widespread pain in schoolchildren: a prospective 4-year follow-up study. Pain, 138(3), 681-687.
27. Mikkelsson, M., Sourander, A., Salminen, J. J., Kautiainen, H., & Piha, J. (1999). Widespread pain and neck pain in schoolchildren. A prospective one-year follow-up study. Acta Paediatr, 88(10), 1119-1124.
28. Mikkonen, P., Heikkala, E., Paananen, M., Remes, J., Taimela, S., Auvinen, J., & Karppinen, J. (2016). Accumulation of psychosocial and lifestyle factors and risk of low back pain in adolescence: a cohort study. Eur Spine J, 25(2), 635-642.
29. Mirovsky, Y., Jakim, I., Halperin, N., & Lev, L. (2002). Non-specific back pain in children and adolescents: a prospective study until maturity. J Pediatr Orthop B, 11(4), 275-278.
30. Mustard, C. A., Kalcevich, C., Frank, J. W., & Boyle, M. (2005). Childhood and early adult predictors of risk of incident back pain: Ontario Child Health Study 2001 follow-up. Am J Epidemiol, 162(8), 779-786.
31. Paananen, M. V., Taimela, S. P., Auvinen, J. P., Tammelin, T. H., Kantomaa, M. T., Ebeling, H. E., . . . Karppinen, J. I. Risk factors for persistence of multiple musculoskeletal pains in adolescence: A two-year followup study. Eur J Pain. 2010 Nov;14(10):1026-32.
32. Perquin, C. W., Hunfeld, J. A. M., Hazebroek-Kampschreur, A. A. J. M., Van Suijlekom-Smit, L. W. A., Passchier, J., Koes, B. W., & Van der Wouden, J. C. The natural course of chronic benign pain in childhood and adolescence: A two-year population-based follow-up study. Eur J Pain. 2003;7(6):551-9.
33. Poussa, M. S., Heliovaara, M. M., Seitsamo, J. T., Kononen, M. H., Hurmerinta, K. A., & Nissinen, M. J. (2005). Predictors of neck pain: a cohort study of children followed up from the age of 11 to 22 years. Eur Spine J, 14(10), 1033-1036.
34. Poussa, M. S., Heliovaara, M. M., Seitsamo, J. T., Kononen, M. H., Hurmerinta, K. A., & Nissinen, M. J. Anthropometric measurements and growth as predictors of low-back pain: A cohort study of children followed up from the age of 11 to 22 years. Eur Spine J. 2005 Aug;14(6):595-8.

35. Salminen, J. J., Erkintalo, M., Laine, M., & Pentti, J. (1995). Low back pain in the young. A prospective three-year follow-up study of subjects with and without low back pain. Spine (Phila Pa 1976), 20(19), 2101-2107
36. Sano, A., Hirano, T., Watanabe, K., Endo, N., Ito, T., Sato, T., . . . Tanabe, N. Body mass index associates with low back pain in childhood and adolescence: A birth cohort study of 6-year follow up in Niigata city, Japan. Eur Spine J, 24(3), 474-481.
37. Sano, A., Hirano, T., Watanabe, K., Endo, N., Ito, T., & Tanabe, N. (2015). Body mass index is associated with low back pain in childhood and adolescence: a birth cohort study with a 6-year follow-up in Niigata City, Japan. Eur Spine J, 24(3), 474-481.
38. Sano, A., Hirano, T., Watanabe, K., Izumi, T., Urakawa, T., Endo, N., . . . Tanabe, N. Low back pain in childhood and adolescence: A longitudinal study of 4-year follow up in Niigata City, Japan. Eur Spine J. 2008 Nov; 17(11): 1441–1447.
39. Siivola, S. M., Levoska, S., Latvala, K., Hoskio, E., Vanharanta, H., & Keinanen-Kiukaanniemi, S. (2004). Predictive factors for neck and shoulder pain: a longitudinal study in young adults. Spine (Phila Pa 1976), 29(15), 1662-1669.
40. Sjolie, A. N., & Ljunggren, A. E. (2001). The significance of high lumbar mobility and low lumbar strength for current and future low back pain in adolescents. Spine (Phila Pa 1976), 26(23), 2629-2636.
41. Stahl, M., Kautiainen, H., El-Metwally, A., Hakkinen, A., Ylinen, J., Salminen, J. J., & Mikkelsson, M. (2008). Non-specific neck pain in schoolchildren: prognosis and risk factors for occurrence and persistence. A 4-year follow-up study. Pain, 137(2), 316-322.
42. Stahl, M., Mikkelsson, M., Kautiainen, H., Hakkinen, A., Ylinen, J., & Salminen, J. J. (2004). Neck pain in adolescence. A 4-year follow-up of pain-free preadolescents. Pain, 110(1-2), 427-431.
43. Szita, J., Boja, S., Szilagyi, A., Somhegyi, A., Varga, P. P., & Lazary, A. (2018). Risk factors of non-specific spinal pain in childhood. Eur Spine J, 27(5), 1119-1126.
44. van Gessel, H., Gassmann, J., & Kroner-Herwig, B. (2011). Children in pain: recurrent back pain, abdominal pain, and headache in children and adolescents in a four-year-period. J Pediatr, 158(6), 977-983.e971-972.
